# Characterization and Genetic Diversity of *Pseudomonas syringae* pv. *syringae* Isolates Associated with Rice Bacterial Leaf Spot in Heilongjiang, China

**DOI:** 10.3390/biology11050720

**Published:** 2022-05-08

**Authors:** Lili Peng, Songrun Yang, Yao Zhang, Haseeb Younis, Shuang Song, Xiaofeng Xu, Mingxiu Yang, Junhua Zhang

**Affiliations:** College of Agriculture, Northeast Agricultural University, Harbin 150030, China; penglili308521@163.com (L.P.); yang1033392001@163.com (S.Y.); zhy92523@126.com (Y.Z.); haseebyounis99@gmail.com (H.Y.); songshuang.000@163.com (S.S.); oxuxiaofeng@163.com (X.X.); 2002ymx@163.com (M.Y.)

**Keywords:** rice, *Pseudomonas syringae* pv. *syringae*, bacteria, multilocus sequence analysis (MLSA), repetitive extragenic palindrome (rep) PCR

## Abstract

**Simple Summary:**

In Northeast China, rice bacterial brown leaf spot caused by *Pseudomonas syringae* pv. *syringae* is among the most damaging rice diseases. This disease results in significant yield losses. This study focuses on a comprehensive analysis of the pathogen, population structure, and genetic diversity within the species based on biochemical tests and genetic characterizations. Our results indicate high genetic heterogeneity in *Pseudomonas syringae* pv. *syringae* isolates, and clustering of testing isolates and reference strains are related with the genomospecies 1. This work contributes to the physiological classification of the *P. s.* pv. *syringae* isolated from Heilongjiang Province, China and the results present new data concerning the phylogeny and genetic diversity. Such studies have not been reported about *P. s.* pv. *syringae* from this region yet.

**Abstract:**

In China, rice is one of the most important cereal crops. Rice bacterial brown leaf spot caused by *P. s.* pv. *syringae* is among the most damaging rice diseases in the Heilongjiang Province of China and results in substantial yield losses. In this study, a comprehensive analysis of the pathogen, population structure, and genetic diversity within the species was performed. For this purpose, 176 bacterial isolates of *P. s.* pv. *syringae* collected from 15 locations were characterized by using biochemical tests such as the LOPAT test, and genetic characterizations such as multilocus sequence analysis (MLSA) and repetitive PCR, using BOX, REP and ERIC primers. Biochemical testing and detection of *syrB* genes confirm the presence of *P. s.* pv. *syringae,* genetic characterization by MLSA and genetic fingerprinting by repetitive PCR confirmed that high genetic heterogeneity exists in the *P. s.* pv. *syringae* isolates, and clustering of the tested isolates and reference strains are related with the same genomospecies 1. This work contributes to the physiological classification of the *P. s.* pv. *syringae* isolated from Heilongjiang Province, China, and the results present new data concerning the phylogeny and genetic diversity. This type of study about *P. s.* pv. *syringae* has been not reported from this region until now.

## 1. Introduction

Bacterial pathogens cause several types of diseases in a wide range of plant species. Many economically important cereal crops are severely affected by bacterial pathogens [1]. The genus *Pseudomonas* consists of several economically destructive pathogens [2]. It includes numerous phytopathogenic bacteria such as *Pseudomonas syringae* (which is Gram-negative), a rod-shaped, aerobic bacterium belonging to the family *Pseudomonadaceae* [3]. The incidence of diseases caused by *Pseudomonas* is among the top ten most spread bacterial plant diseases. It can infect Legumes, Cruciferae, Solanaceae, Rosaceae, and more than three hundred crops. *Pseudomonas syringae* pv. *syringae* causes bacterial leaf spot which is a widespread disease. This pathogen affects rice, tobacco, parsley [4], green pumpkin [5], fig tree, kiwi fruit, and philodendron [6]. It has caused huge losses to agricultural production.

Heilongjiang is one of the major rice-producing provinces of China. Rice is the main food crop and its planting area reached almost 3.87 million hectares in 2020. Rice bacterial brown leaf spot caused by *Pseudomonas syringae* pv. *syringae* is among the most destructive rice disease of Heilongjiang Province of China. According to data obtained from the Plant Quarantine and Protection station, the disease-affected area reached up to 211,000 hectares in the Heilongjiang Province. Symptoms of the disease appear as brown water-stained spots, with yellow halo margins in the early stage, and spindle-shaped, oblong, or irregular-shaped lesions, gray in the center, in the late stage. In severe cases, partial leaves die.

This disease was first reported in Hungary by Klement. This disease attacks the leaves and sheathes the panicles of rice. The bacteria causing this disease is named *Pseudomonas oryzicola* [7]. This species is a synonym of *P. s.* pv. *syringae* [8]. Fang and Ren [9], and Khu and Bai [10], also reported this disease in the Eastern and Northeastern provinces of China. A brown leaf spot caused by a similar bacterium, but further investigation revealed that it formed acid from maltose, weakly hydrolyzed starch, and had a smaller size, which is different from previously reported *Pseudomonas* later in Japan. Funayama and Hirano reported this disease causing brown discoloration of the leaf sheath in rice as *P. s.* pv.*syringae* [10]. Under cool and humid conditions, *P. s.* pv. *syringae* caused more damage and it spread more quickly and easily. It also attacked 30 species of cultivated and wild Gramineae [11]. It is possible that several organisms are involved which have similar disease symptoms and genetic diversity within these species also needs investigating. In northeast China, this type of disease is caused by *P. s*. pv. *syringae*. So, the genetic diversity within this species needs investigation. Regardless of the *P. s.* pv. *syringae* occurrence in rice-growing regions of Heilongjiang Province, the lack of genetic and molecular studies about *P. s.* pv. *syringae* variation is the main aspect that restrict the understanding of pathogens. An improved understanding of the population structure and epidemiology of pathogens can help to predict and develop suitable models of disease outbreaks. To achieve this purpose, *P. s.* pv. *syringae* isolates were collected from 15 main rice-producing areas of Heilongjiang Province, China.

Molecular tools, such as sequencing, polymerase chain reaction (PCR), loop-mediated isothermal amplification, and multilocus sequence analysis (MLSA), were vital for species identification [12,13]. Molecular characterization and genetic diversity of pathogen populations are critical to understanding the dynamics of pathogen populations to develop effective strategies for disease outbreaks.

In this experiment, *P. s.* pv. *syringae* isolates were studied by partial 16S rRNA sequencing [14,15] and *syrB* analysis to determine the toxin involved in *P. s.* pv. *syringae* virulence [16], and multilocus sequence analysis (MLSA) [4]. Additionally, the pathogenic bacteria were characterized further by using enterobacterial repetitive intergenic consensus (ERIC, BOX and ERIC). Moreover, a pathogenicity test was also performed. Therefore, the major aims of this study were to reveal the potential genotypic variability and to determine the relatedness of *P. s.* pv. *syringae* strains. In this study, we identified the genetic diversity and characterize the *P. s.* pv. *syringae* isolates collected from Heilongjiang Province, China.

## 2. Materials and Methods

### 2.1. Sample Collection and Bacterial Isolates

The infected rice plants were collected from 15 main rice-producing areas of Heilongjiang Province, China (Table S1). These rice plants present disease symptoms of brown spots and necrotic lesions from the leaf tips and margins. First, a small piece of leaf tissue was disinfected with 70% ethyl alcohol, then in 1% sodium hypochlorite, leaf tissue was dipped for 1 min and washed with sterile distilled water. Then, leaf tissue was cut and crushed using a pipette tip in a 1.5 mL tube with 100 µL sterile water. On King’s B medium, the suspensions were streaked [17]. After incubation incubation of 2 days at 26 ℃, a total of 176 colonies were purified and selected on peptone sucrose and frozen at -80 ℃ in 50% glycerol.

### 2.2. Biochemical Characteristics

Biochemical characteristics of bacterial isolates were analyzed by standard bacteriological methods such as Gram-staining, hydrolysis gelatin, nitrate reductase, LOPAT (levan production, oxidase reaction, potato soft rot, arginine dihydrolase activity, and tobacco hypersensitivity) tests [18], and utilization of various carbon sources. These tests were accomplished according to the method described by Schaad et al. [19]. 

### 2.3. Pathogenicity Test

Pathogenicity tests of isolates were executed by inoculation of the bacterial pathogen on rice leaves according to the described methods by Lelliott and Stead [18], and Klement et al. [20]. On Luria Pepton (LP) (Difco) medium, all the strains were regrown for 2 days at 28 ℃. In sterile distilled water, the bacterial suspensions were prepared and the final concentration of 10^8^ cfu/mL was adjusted photometrically. All 176 strains were tested for pathogenicity and each strain was tested and inoculated on two rice plants in triplicate. All the plant was inoculated with a diluted bacteria suspension through the spray method, sterile water was used as a control. In plastic bags, inoculated plants were covered and incubated at 25 ℃ for 10 days. Pathogenic reactions were estimated 10 days after the inoculation. In addition, the susceptibility of the strains through copper sulphate (CuSO_4_) and streptomycin were also determined. 100 µL of the bacterial suspensions (10^8^ cfu/mL) was spread on the nutrient agar (NA) medium with 50, 100, 200, 400 mg/L CuSO_4_ and 0 mg/L CuSO_4_ as a negative control. For streptomycin, 100 µL of the bacterial suspensions (10^8^ cfu/mL) was spread on the nutrient agar (NA) medium with 20, 50, 100, 200 mg/L streptomycins and 0 mg/L streptomycins as a negative control.

### 2.4. DNA Extraction

From purified bacterial cultures, DNA was extracted, following the manufacturer’s instructions on Bacteria Genomic DNA Kit (CWBIO, Beijing, China). NanoDrop™ 2000/c spectrophotometer (Thermo Fisher Scientific, Waltham, MA, USA) was used to compute the isolated DNA. For further investigation, this extracted DNA was stored at -80 ℃. 

### 2.5. syrB Gene Detection

In all the tested isolates, *syrB* gene was detected to confirm the *Pseudomonas syringae pv. syringae*. The primer pair was used to detect *syrB* gene described in Table S2. According to the described method by Sorensen et al. [16], PCR was amplified. It was programmed for 35 cycles, primer annealing at 60 ℃ for 90 sec, denaturation at 94 ℃ for 90 sec, DNA extension for 3 min at 72 ℃ and further extension at 72 ℃ for 10 min. PCR reactions were performed in 20 µL volumes in a T100 Thermocycler (Bio-Rad, Hercules, CA, USA). 

### 2.6. Phylogenetic Analysis of 16S rRNA

For molecular identification, universal primers B1 and B2 (Table S2) amplicons for the partial 16S rRNA sequences were produced by PCR. The reaction conditions for PCR are described by Scortichini et al. [21]. From agarose gel, products were purified and sequenced by Sangon Biotech (Shanghai, China). Sequences were deposited in the GenBank database. To match the unidentified sequences with sequences in public databases, a basic alignment search tool, BLAST, was used [22]. Multiple alignments and comparisons with reference strains were carried out by using CLUSTALW integrated into MEGA 5.0 software. Phylogenetic analyses were accomplished by using the neighbor-joining (NJ) method.

### 2.7. Multilocus Sequence Analysis

Relationship between the *P. s.* pv. *syringae* isolates collected from Heilongjiang Provence and reference strains of *P. syringae* were determined by multilocus sequence analysis (MLSA). Three housekeeping genes such as *rpoD*, *gyrB*, and *gltA* were amplified from genomic DNA and sequenced with primer pairs described in Table S2. The following program was used to perform PCR reaction: 3 min at 94 °C, 35 cycles of 30 sec at 94  °C, 30 sec at 56  °C, 72 °C for 30 sec and final elongation at 72 °C for 10 min. From agarose gel, products were purified and sequenced by Sangon Biotech (Shanghai, China). Sequences were deposited in the GenBank database. Sequences were edited and aligned using MEGA 5.0 and matched with other *Pseudomonas* sp. sequences by using BLAST.

### 2.8. Repetitive PCR

Genetic fingerprinting of *P. syringae* isolates with reference strains was performed by repetitive sequence-based PCR (rep-PCR) using the BOX-PCR, ERIC-PCR [23,24]. Repetitive PCR (REP-PCR) was performed by using the genomic DNA from each strain and the primer sets were described in Table S2. Repetitive PCR (REP-PCR) amplification is carried out by following cycles: 1 cycle for 7 min at 95 °C; 34 cycles for 1 min at 94 °C of denaturation, annealing for 1 min at 40 °C (53 °C in BOX-PCR, 52 °C in ERIC-PCR), and extension for 8 min at 65 °C with a single final extension cycle for 15 min at 65 °C and a final immersion at 4 °C. PCR amplifications were repeated three times. To separate amplified PCR products, gel electrophoresis was performed on 2 % agarose gels in 1× TAE buffer for 1.5 h at 5 V/cm, stained with 0.05 µL/mL ethidium bromide and visualized under UV illumination. Generated fingerprints of isolated strains were optically compared.

### 2.9. Data Analysis

Genotypic profiles produced by rep-PCR were assessed by the BioNumerics V7.6 software (Applied-Maths, Sint-Martens-Latem, Belgium), using the Dice coefficient for pairwise comparison, and the unweighted pair group method, using arithmetic averages (UPGMA) clustering [25].

## 3. Results

### 3.1. Identification and Characterization of Bacterial Strains

Rice plants were collected from several fields with suspected bacterial disease symptoms from 15 main rice-producing areas of Heilongjiang Province, China (Figure S1). The pathogen had been isolated from plant tissues that had typical disease symptoms. A sum of 176 bacterial strains were grown and purified on NA medium (Table S1). All of the strains were selected for biochemical characterization (Table 1). Their results showed that the morphological characteristics of 176 strains were the same. These strains were grown at 27 ℃ for 48 h, and they showed a white circular colony of 2–3 mm diameter. The consequences of biochemical characterization of 176 strains were consistent. For bacterial characterization, different biochemical tests were performed. The results of these tests are as follows: levan formation, tobacco hypersensitivity, and catalase exhibit positive reactions, but potato soft-rot, oxidase, and hydrolysis gelatin and growth at 41 ℃ have a negative reaction (Table 1). Some other biochemical tests such as arginine dihydrolase, glucose, mannitol, and inositol also showed positive reactions which indicate that the strain has classic characteristics of *P. s.* pv. *syringae*.

### 3.2. Pathogenicity Tests

**Figure 1:**
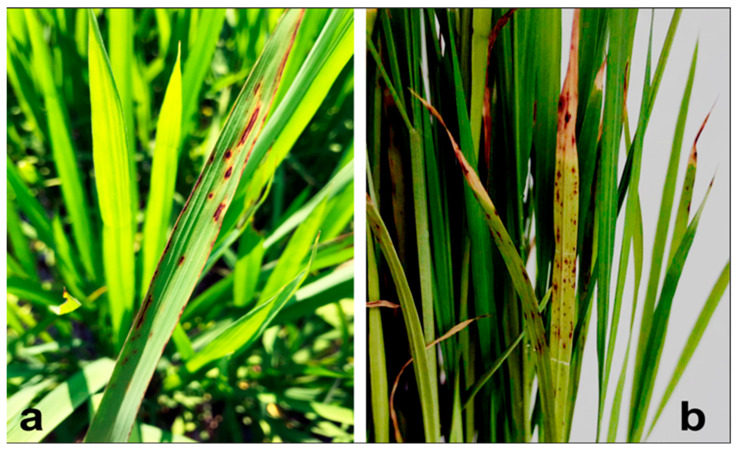
Field symptoms of rice bacterial brown spot diseases for the whole plant (**a**) and inoculated leaves (**b**).

To conduct pathogenicity tests, all inoculated rice plants showed disease symptoms after 10–14 days of bacterial inoculation. Initially, they showed water-soaked lesions, which gradually turned into chlorotic spots over time (Figure 1b). Control plants remained healthy and had no disease symptoms. In addition, it indicated that all the tested strains were susceptible to all concentrations of streptomycin and non-susceptible to copper sulphate at the concentration of 100 mg/L.

### 3.3. Molecular Characterization

*P*. *s.* pv. *syringae* produce a toxin called syringomycin. The *syrB* gene is responsible for producing syringomycin, so *P*. *s.* pv. *syringae* can be detected by these genes. In all tested 176 isolates, *syrB* gene was amplified by PCR with primers *syrB*-F and *syrB*-R. These primers amplify *syrB* only in *P. s.* pv. *syringae*. At the 752 bp band, the *syrB* gene was amplified. All the strains were successfully amplified and hence possess the *syrB* gene. The sequence analysis of the *syrB* gene was deposited in the NCBI GenBank database under their specific accession numbers MK453195, MK453196, MK453197, MK453198, and MK453199 for five selected samples (Table S3). 

**Figure 2:**
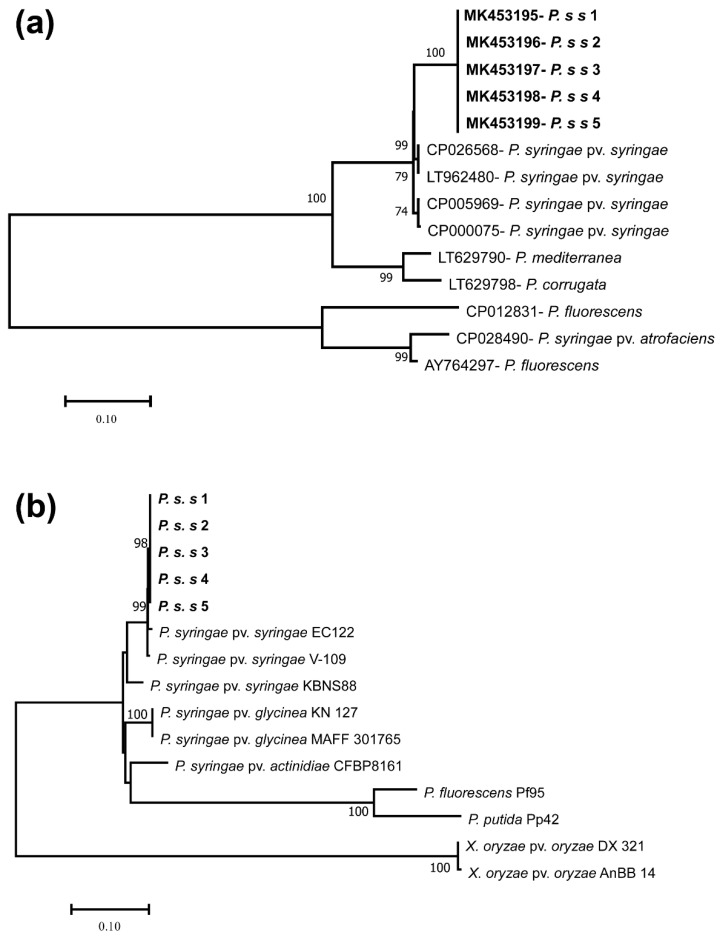
Joining phylogenetic trees neighbor based on *syrB* (**a**) and 16S rRNA (**b**).

The 16S rRNA gene-sequencing technique was used to classify bacteria at the species level. The accession number of 16S rRNA for five selected *P*. *s.* pv. *syringae* samples are MT256188, MT256107, MT256183, MT256185, and MT256186. Results of both the *syrB* tree (Figure 2a) and 16S rRNA tree (Figure 2b) showed that clusters of *P.s.s* 1 to *P.s.s* 5 selected earlier, together with the isolates of *P. s.* pv. *syringae,* have a high percentage of bootstrap values. It is confirmed that *P. s.* pv. *syringae* isolates collected from rice plants in Heilongjiang Province, China showed 100% homology with *P. s.* pv. *syringae* strain *Pss*9097 (accession number CP026568) and IPPBC-R30 (accession number HQ840766) from NCBI GenBank (Figure 2). Therefore, it can be concluded that the isolates examined in this study can be recognized as *P. s.* pv. *syringae.*

**Figure 3:**
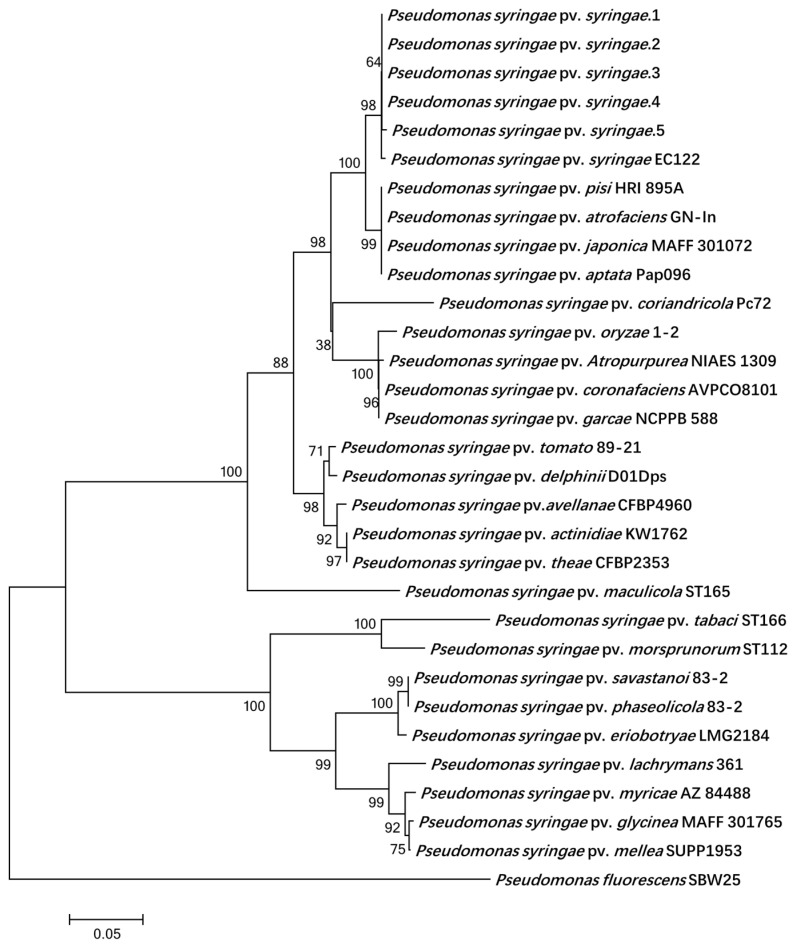
Neighbor-joining phylogenetic tree of *P. s.* pv. *syringae* strains from rice and reference strains derived from a concatenation of *gyrB*-*rpoD*-*gltA* genes. Bar—estimated nucleotide substitutions per site is 0.05.

The sequences generated with three housekeeping genes (*rpoD*, *gyrB*, and *gltA*) and NJ-tree-constructed data showed that *P.s.s* 1 to *P.s.s* 5 were gathered with the following reference of strains from the database: *P. syringae* pv. *pisi* HRI 895A, *P. syringae* pv. *atrofaciens* GN-In, *P. syringae* pv. *japonica* MAFF 301072, *P. syringae* pv. *aptata* Pap096 and *P. s.* pv. *syringae* EC122 (Figure 3). The sequences and accession numbers of *P.s.s*1 to *P.s.s*5 for three housekeeping genes (*rpoD*, *gyrB*, and *gltA*) were deposited in the GenBank (Table S3). From MLSA, a phylogenetic tree was obtained, showing that our isolates were associated with genomospecies 1 of *P. syringae* [26]. 

### 3.4. Rep-PCR Analysis

**Figure 4:**
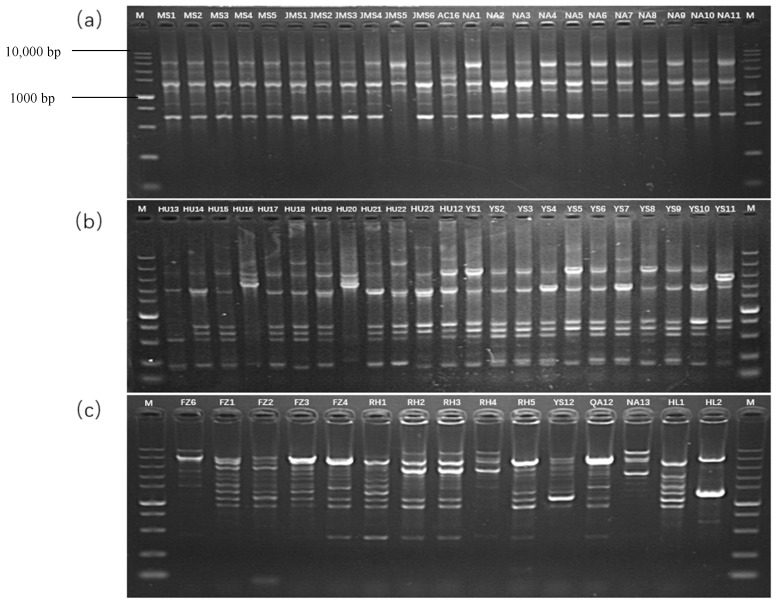
Genomic fingerprints of *P. s.* pv. *syringae* isolates generated by BOX (**a**), ERIC (**b**), and REP (**c**) primers in 2% agarose gel.

REP-PCR genomic fingerprints were produced to examine the genetic diversity of 176 *P. s.* pv. *syringae* isolates. Banding patterns of DNA were multifarious among tested isolates and showed genetic diversity (Figure 4). By using the fingerprinting technique, distinct banding patterns were generated. Oligonucleotide primers and the different PCR situations were used in this process. In BOX-PCR analysis, the amplified band’s length ranged from 500 to 3000 bp (Figure 4a), and ERIC and REP-PCR ranged from 100 to 10,000 bp (Figure 4b,c).

Reproducible fingerprint profiles were produced with each technique by repeating the procedures. For the combination of rep-PCR results, a dendrogram was calculated by Dice coefficients with unweighted pair group method clustering (UPGMA), based on 37.5% similarity level (Figure S2); strains were divided into eight main groups and different colors represent 15 different regions of Heilongjiang Province. Cluster Ⅰ includes 19 *P. s.* pv. *syringae* isolates from the Suiling and 3 *P. s.* pv. *syringae* isolates from Acheng and Ningan region, with cluster Ⅱ largely consisting of isolates from the Yanshou region and a small portion of the Xingkaihu Farm and only one isolate from the Qing an, Mingshui and Hailun regions, and all the strains isolated from Raohe and Fangzheng region; cluster Ⅲ includes 14 isolates from five different regions. Cluster Ⅳ is the most diverse cluster consisting of 50 isolates from nine different regions, this cluster also has the highest number of strains. Cluster V consists of 39 isolates from five regions; it consists of all the strains isolated from Wuchang, Jiamusi and Qianjin. However, groups Ⅵ, Ⅶ and Ⅷ have the lowest number of strains: they only have three, three, and one strain, respectively.

**Figure 5:**
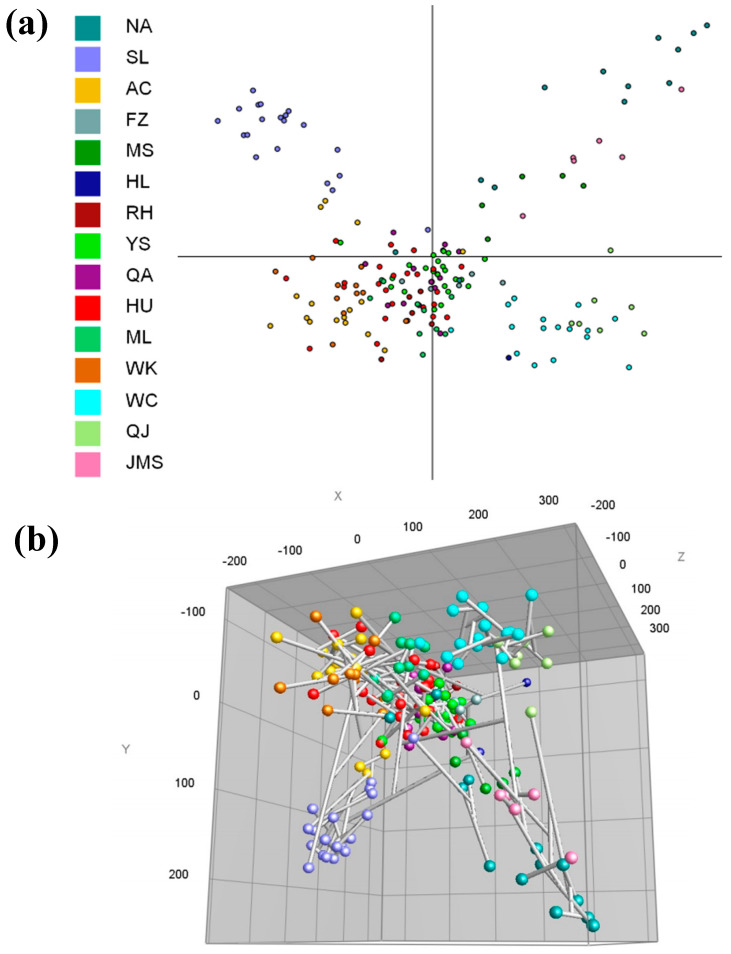
Principal coordinates analysis (PCoA) plots produced from *P. s.* pv. *syringae* profiles identified from the 15 studied regions using BioNumerics V7.6 software Plan view (**a**). An ideal combination for joint evaluation of tree graphs and PCoA (**b**).

To establish a potential correlation between the regions considering *P. s.* pv. *syringae* genetic profile diversity, a principal component analysis (PCA) was performed. This analysis enables us to correlate the *P. s.* pv. *syringae* population and diversity of each region of Heilongjiang Province; *P. s.* pv. *syringae* populations from Muling, Qing an, Xingkaihu Farm, Acheng and Wangkui were related and distinct from those found in the Jiamusi and Mingshui region (Figure 5a). This configuration was mainly owing to the high abundance of ML, QA, HU, AC and WK strains in these regions when compared with the lesser abundance in other regions. In addition, *P. s.* pv. *syringae* populations from Wuchang and Qianjin were highly correlated (Figure 5b). *P. s.* pv. *syringae* populations from Suiling were different from other populations. From the results, we can conclude that there is no direct relation between the genetic relationship and geographical distance. The genetic relationship between two regions located far away from each other may be very close. Similarly, the genetic relationship between two closely located regions may be different.

## 4. Discussion

The incidence of bacterial brown leaf spot in rice caused by *P. s.* pv. *syringae* in Heilongjiang Province of China was reported by many scientists but a comprehensive study of the pathogen population structure and genetic diversity within the species was not conducted until now. *P. s.* pv. *syringae* is a widespread disease. This pathogen infects numerous plant species such as citrus, apples, pear, Ficus carica L. (Ivanović, Ž) [6] and Actinidia deliciosa (A. Chev.) C.F. Liang and A.R. Ferguson (Ivanović, Ž and Balestra, G.M.) [6,27]. *P. s.* pv. *syringae* spread rapidly in cool and moist conditions and this pathogen can easily enter through wounded and damaged plant tissue by winds or cool temperatures.

In our study, 176 isolates originating from 15 main rice-producing areas of Heilongjiang Province were investigated. These strains were characterized and identified by using biochemical and molecular tests. Results of these tests confirm the *P. s.* pv. *syringae* presence. Biochemical characteristics of bacterial isolates were analyzed by standard bacteriological methods such as Gram-staining, hydrolysis gelatin, nitrate reductase, LOPAT and utilization of various carbon sources. Characteristics of representative strains of bacterial isolates indicated that it was a Gram-negative bacteria, with a maximum death temperature of 41 °C, negative for hydrolyzed gelatin, nitrate reductase, oxidase activity, potato rot, and positive for fructan , arginine and tobacco hypersensitivity. In terms of carbon source utilization, glucose, lactose, mannose and sucrose can be used, but maltose and trehalose can not be used. All of the results indicated typical *P. s.* pv. *syringae. P. s.* pv. *syringae* strains have a unique character of producing and export of syringomycins and syringopeptins toxins; *syrB* and *syrD* genes are accountable for the creation of these toxins, respectively [5]. All the strains isolated from rice have the *syrB* gene which is a toxin-producing gene. It indicates that the pathogen that causes the rice bacterial brown leaf spot is *P. s.* pv. *syringae.* Genetic characterizations such as multilocus sequence analysis (MLSA) and 16S rRNA gene sequencing further confirm that the phylogenetic tree obtained from 16S rRNA and MLSA shows a phylogenetic relationship with *P. s.* pv. *Syringae*; all of the *P. s.* pv. *syringae* isolates belong to the genomospecies 1 of *P. syringe* [26]. The genetic variability in the *P. s.* pv. *syringae* isolates was evaluated by rep-PCR using BOX, REP, and ERIC primer sets. Their patterns were analyzed with UPGMA and Dice’s coefficients [25]. Based on a 37.5% similarity level, strains were divided into eight main groups and there is no direct relation between the genetic relationship and geographical distance indicated by principal component analysis (PCA).

This study indicates that there is significant genetic heterogeneity within the *P. s.* pv. *syringae*. The variability was consistent with earlier studies utilizing ERIC-PCR [28], or BOX-PCR [29,30], on the matching strains or ERIC-PCR [31], or AFLP analyses [32,33], on additional *P. s.* pv. *syringae* strains. Based on genetic and phenotypic confirmation, a survey shows that *P. s.* pv. *syringae* isolates from heart-leaf philodendron, the fig tree and kiwi fruit were similar on ERIC and REP-PCR, but with marginally different genetic DNA profiles on BOX-PCR [6]. However, the fingerprints of 176 isolates of rice from 15 different regions showed no significant difference on BOX-PCR, but there was significant diversity on ERIC-PCR and REP-PCR (Figure 4). Therefore, in the analysis of our research, the results obtained by BOX, ERIC and REP were jointly analyzed to obtain the final cluster diagram, which showed that the pathogen of P. s. pv. syringae isolated from rice of Heilongjiang Province in China had high genetic heterogenity and there was no obvious correlation with geographic location. However, most of the strains classified into the second group in the cluster diagram are from Yanshou, Xingkaihu Farm, Raohe and Fangzheng strains (Figure S2). The map of Heilongjiang Province in Figure S1 shows that these four regions are relatively close in terms of geographical distance, which are located in the southeastern region of Heilongjiang Province. It indicate that the population of *P. s.* pv. *syringae* may have different degrees of bacterial source communication between different locations in the same region. The reason may be that strains from the same geographical area face the same selection pressure under the same geographical conditions, which leads to directional selection of their genetic material. However, the strains isolated from two regions with relatively close geographical distance, such as Suiling and Hailun, have a farther genetic relationship. It may be that the strains at the two sites face different selection pressures due to differences in soil and climatic conditions, and their genetic material makes directional selection. The results of the conjoint analysis would provide stronger evidence. Differences in climatic factors, epidemic dynamics, and disease control measures probably contributed to the extensive genomic diversification of *P. s.* pv. *syringae,* which is responsible for bacterial brown leaf spot of rice in Heilongjiang Province, China. This type of study about *P. s.* pv. *syringae* has not been reported from this region. These results provide an primary position to study genetic connection among the strains of rice bacterial brown leaf spot originating from different regions of Heilongjiang.

The pathogen of *P. s.* pv. *syringae* can spread in numerous ways such as aerial, dust, or rain splashes and its attack on different hosts is not specific to one species [33,34,35]. This disease has a serious economic effect on rice. In a previous study conducted in Iran, the isolated strains, which cause foot and sheath rot of rice, were divided into two groups based on physiological, morphological and biochemical features, pathogenicity, and PCR with specified primers [36]. The first group consisted of 21 strains of *P. c. sub* sp. *ca rotovorum*, which produced foot rot. The second group consisted of 22 *P. s.* pv. *syringae* strains that induced sheath rot. There were no significant variations in the severity of illness and symptoms. These findings show that the pathogenicity of bacteria obtained from diverse fields is similar. So, study of the biological control of bacterial brown leaf spot on rice by antagonistic strains, pathogenicity testing in different parts of Heilongjiang Province of China, and use of resistant cultivars could be a case study for future research.

## 5. Conclusions

Rice bacterial brown leaf spot is one of the most damaging rice diseases in Heilongjiang Province of China. This study provides a comprehensive analysis of the pathogen, population structure, and genetic diversity within the species based on biochemical tests and genetic characterizations. Phylogenetic analyses of *gyrB*, *rpoD*, and *gltA* gene sequences showed that *P. syringae* pv. *syringae* causes rice bacterial brown leaf spot, and belongs to genomospecies 1. The results also indicate high genetic heterogeneity in *P. s.* pv. *syringae* isolates by rep-PCR. This work presents new data concerning the phylogeny and genetic diversity. Such studies have not been reported about *P. s.* pv. *syringae* from this region yet, and this work contributes to further epidemiological studies of this pathogen and for determining breeding strategies for *P. s.* pv. *syringae* control.

## Figures and Tables

**Table 1:** Origin and main biochemical characteristics of *P. s.* pv. *syringae* isolates.

Characteristics		Reaction
Growth at 41 °C		−
Detection of *syrB*		+
Gram staining		−
Hydrolysis gelatin		−
Nitrate reductase		−
LOPAT Test	Levan formation	+
	Oxidase	−
	Potato rot	−
	Arginine dihydrolase	+
	Tobacco hypersensitivity	+
Utilization as carbon source	Glucose	+
	Mannose	+
	Lactose	+
	Maltose	−
	Sucrose	+
	Trehalose	−

Symbol of “+” represents positive response for the tested strain, and “−” represents negative response for the tested strain.

## Data Availability

The data presented in this study are available on request from the corresponding author. The data are not publicly available due to privacy.

## Supplementary Materials

The following are available online at https://www.mdpi.com/article/10.3390/biology11050720/s1, Figure S1: The 15 main rice producing areas in Heilongjiang Province of China, Figure S2:Clustering of *Pseudomonas syringae* pv.*syringae* isolates from *Oryza sativa* L. Table S1: Total isolates of *Pseudomonas syringae* pv.*syringae* strains in this study, Table S2: Tests and list of primers used in this study, Table S3: Reference strains for detection of toxin-producing and MLSA analysis.
