# Supplementary material for: Characterization and Genetic Diversity of Pseudomonas syringae pv. syringae Isolates Associated with Rice Bacterial Leaf Spot in Heilongjiang, China
Source: Biology (Basel). 2022 May 8;11(5):720. doi: 10.3390/biology11050720 (PMC9138427; doi:10.3390/biology11050720)
Supplement: Supplementary file 1 [file biology-11-00720-s001.zip › biology-1637790-supplementary.pdf]

Table S1. Total isolates of *Pseudomonas syringae* pv.*syringae* strains in this study.

Strain	Location	Host	Number	Isolation
MS1~5	Mingshui	*Oryza sativa* L.	5	2018，2019
QA1~12	Qing an	*Oryza sativa* L.	12	2018，2019
JMS1~6	Jiamusi	*Oryza sativa* L.	6	2018，2019
YS1~24	Yanshou	*Oryza sativa* L.	24	2018，2019
ML1~10	Muling	*Oryza sativa* L.	10	2018，2019
SL1~20	Suiling	*Oryza sativa* L.	20	2018，2019
HU1~23	Xingkaihu Farm	*Oryza sativa* L.	23	2018，2019
WC1~16	Wuchang	*Oryza sativa* L.	16	2018，2019
RH1~5	Raohe	*Oryza sativa* L.	5	2018，2019
AC1~16	Acheng	*Oryza sativa* L.	16	2018，2019
WK1~12	Wangkui	*Oryza sativa* L.	12	2018，2019
NA1~13	Ning an	*Oryza sativa* L.	13	2018，2019
QJ1~7	Qianjin	*Oryza sativa* L.	7	2018，2019
FZ1~5	Fangzheng	*Oryza sativa* L.	5	2018，2019
HL1~2	Hailun	*Oryza sativa* L.	2	2018，2019
Total Number		176		

Table S2. Tests and list of primers used in this study.

Primer	Sequence (5'-3')	Amplicon size	Reference
gltA-F	AGTTGATCATCGAGGGCGCWGCC	600 bp	Sarkar&Guttma, 2004
gltA-R	TGATCGGTTTGATCTCGCACGG		
rpoD-F	AAGGCGARATCGAAATCGCCAAGCG	612 bp	Sarkar&Guttma, 2004
rpoD-R	GGAACWKGCGCAGGAGTCGGCACG		
gyrB-F	MGGCGGYAAGTTCGATGACAAYTC	516bp	Sarkar&Guttma, 2004
gyrB-R	TRATBKCAGTCARACCTTCRCGSGC		
syrB-F	CTTTCCGTGGTCTTGATGAGG	752bp	Sorensen, 1998
syrB-R	TCGATTTTGCCGTGATGAGTC		
16s rRNA-B1	CTTTCCGTGGTCTTGATGAGG	-	Scortichini,2005
16s rRNA-B2	TCGATTTTGCCGTGATGAGTC		
ERIC1R	ATGT AAGCTCCTGGGGATTCAC	-	Lupski, 1992
ERIC2	AAGTAAGTGACTGGGGTGAGCG		
REP1R-I	IIIICGICGICATCIGGC (I= inosine)		
REP2-I	ICGICTTATCIGGCCTAC (I= inosine)		
BOX	CTACGGCAAGGCGACGCTGACG		

Table S3. Reference strains for detection of toxin-producing and MLSA analysis.

reference strains	Location	Host	16s rRNA	*syrB*	*rpoD*	*gltA*	*gyrB*
P.s.s 1	Wangkuis	*Oryza sativa* L.	MT256188	MK453195	MK453210	MK453200	MK453205
P.s.s 2	Fangzheng	*Oryza sativa* L.	MT256107	MK453196	MK453211	MK453201	MK453206
P.s.s 3	Wuchang	*Oryza sativa* L.	MT256183	MK453197	MK453212	MK453202	MK453207
P.s.s 4	Yanshou	*Oryza sativa* L.	MT256185	MK453198	MK453213	MK453203	MK453208
P.s.s 5	Acheng	*Oryza sativa* L.	MT256186	MK453199	MK453214	MK453204	MK453209

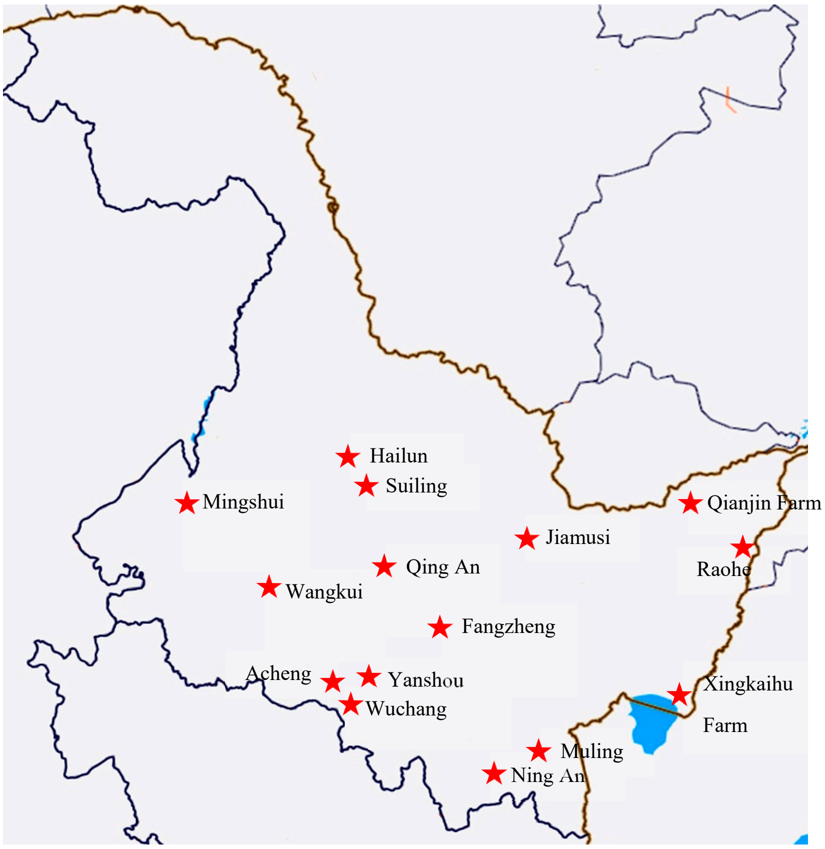

Figure S1. The 15 main rice-producing areas in Heilongjiang Province of China.

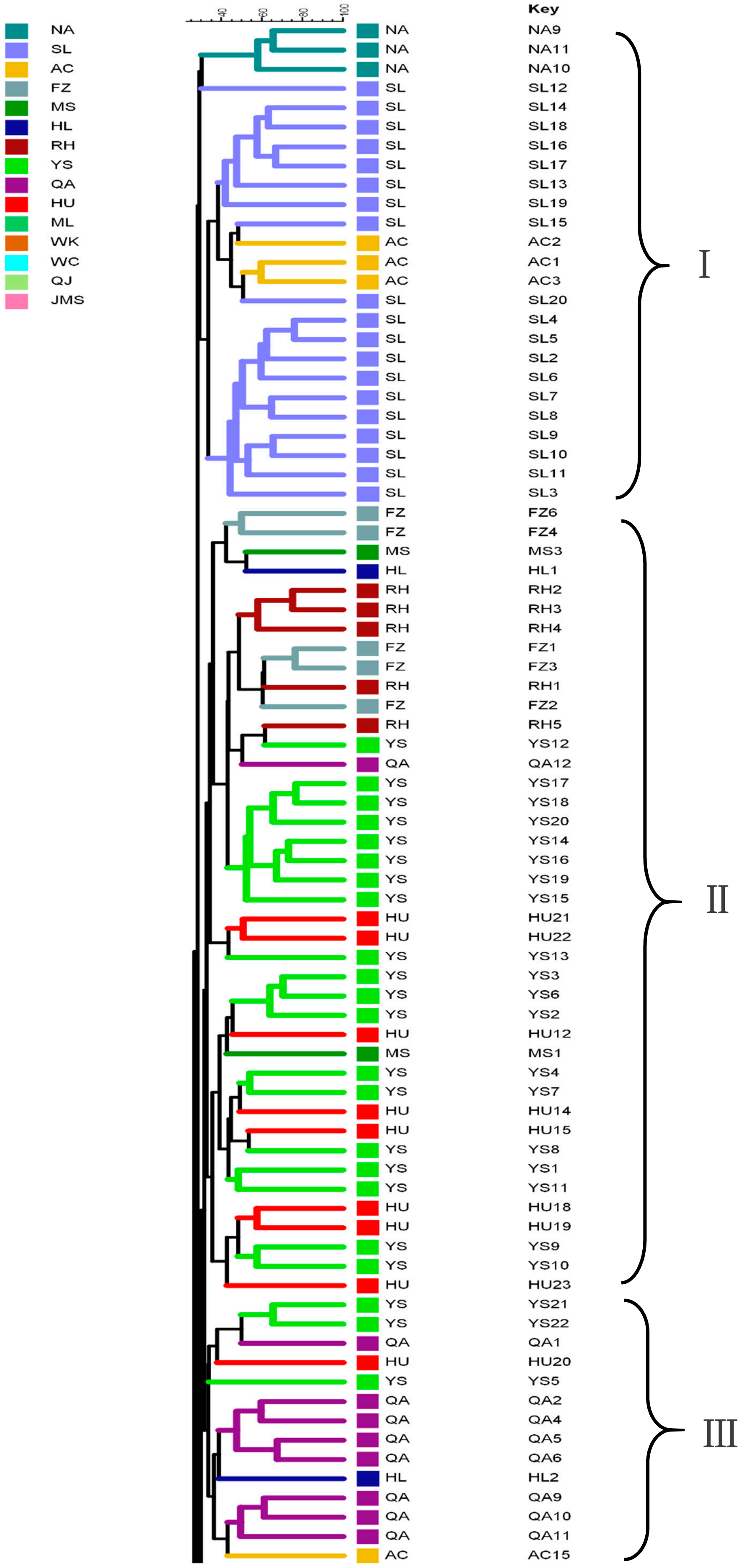
NA
SL
AC
FZ
MS
HL
RH
YS
QA
HU
ML
WK
WC
QJ
JMS
40
60
80
100
Key
NA9
NA11
NA10
SL12
SL14
SL18
SL16
SL17
SL13
SL19
SL15
AC2
AC1
AC3
SL20
SL4
SL5
SL2
SL6
SL7
SL8
SL9
SL10
SL11
SL3
FZ6
FZ4
MS3
HL1
RH2
RH3
RH4
FZ1
FZ3
RH1
FZ2
RH5
YS12
QA12
YS17
YS18
YS20
YS14
YS16
YS19
YS15
HU21
HU22
YS13
YS3
YS6
YS2
HU12
MS1
YS4
YS7
HU14
HU15
YS8
YS1
YS11
HU18
HU19
YS9
YS10
HU23
YS21
YS22
QA1
HU20
YS5
QA2
QA4
QA5
QA6
HL2
QA9
QA10
QA11
AC15
I
II
III

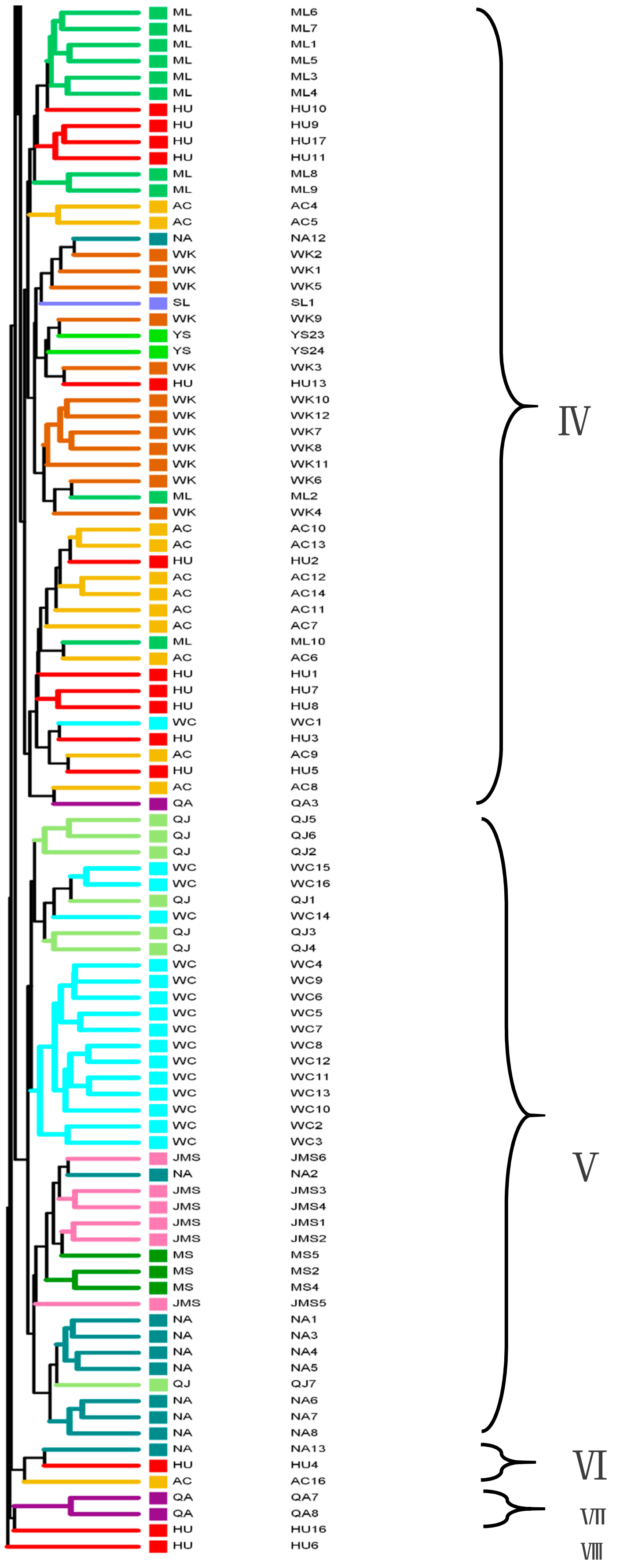

Figure S2. Clustering of *Pseudomonas syringae* pv.*syringae* isolates from *Oryza sativa* L.
